# The conserved microtubule-associated protein Mini Spindles promotes dendrite branching and limits terminal branch elongation in *Drosophila* class III and IV dendritic arborization neurons

**DOI:** 10.17912/micropub.biology.001056

**Published:** 2026-02-19

**Authors:** Claire Kittock, Noor Anvery, Mala Misra

**Affiliations:** 1 University of South Dakota Sanford School of Medicine; 2 SUNY Downstate Medical Center, Brooklyn, New York, United States; 3 Department of Biology, Washington College, Chestertown, Maryland, United States

## Abstract

Microtubule dynamics influence neuron morphogenesis. We investigated the role of the conserved microtubule-associated protein Mini Spindles (Msps) in the morphogenesis of branched dendrite arbors using *Drosophila melanogaster* larval class III and class IV dendritic arborization neurons as two models of branch organization. In both classes, knocking down *msps* expression reduced dendrite branching but increased terminal dendrite length. In *msps ^RNAi^* class IV da neurons, dendrite growth failed to scale in proportion to increasing larval size between the second and third instar. These results suggest that Msps is required for the dynamic expansion of dendrite arbors during periods of rapid organismal growth.

**Figure 1. Loss of msps causes reduced dendrite branching and elongated terminal dendrites:**
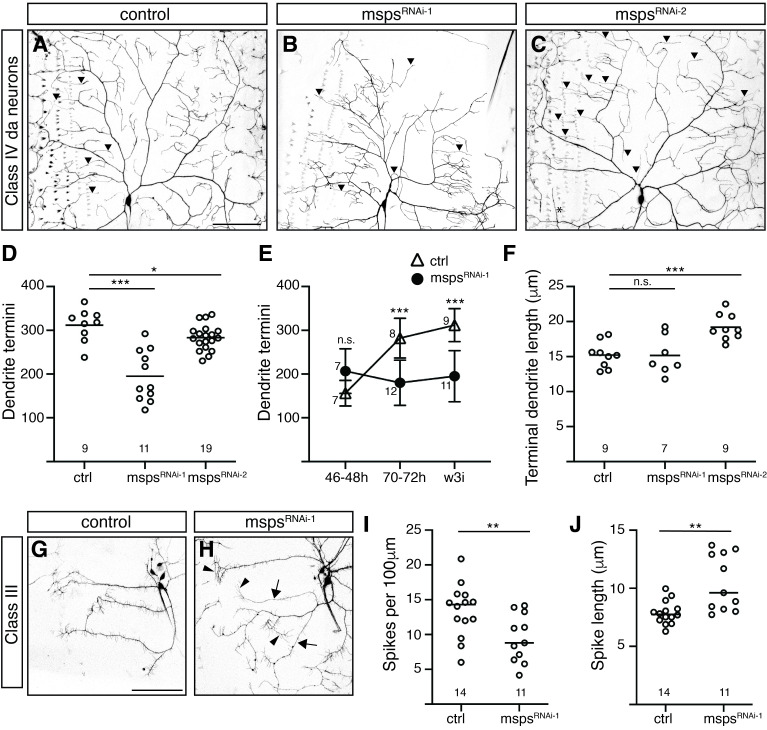
A-C. GFP-labeled ddaC neurons from abdominal segment A2 or A3 in live *ppk-GAL4>* control ( *yw* ), > *UAS-msps ^RNAi-1^* , and > *UAS-msps ^RNAi-2^* larvae. Ultra-long terminal dendrites (length greater than 3 *θ* from the mean length of control dendrites) are indicated with black triangles. D-E. Quantification of terminal dendrite numbers in ddaC neurons from A2 and A3 of 46-48h AEL, 70-72h AEL, and wandering third instar (w3i) larvae. F. Quantification of average terminal dendrite length. G-H. GFP-labeled ddaA and ddaF neurons from A2 or A3 in live *1003.3-GAL4>* control ( *yw* ) and > *UAS-msps ^RNAi-1^* larvae. Images include regions from four adjacent image tiles; because tiles were captured in series and live larvae may shift during imaging, some regions appear offset. Arrowheads indicate regions of increased spike length in *msps ^RNAi-1^* arbors; arrows indicate regions of diminished spike density compared to controls. I. Quantification of spikes per 100μm of primary dendrite length. J. Quantification of average spike length. In D and I, circles represent the absolute number of termini or the spike density in a neuron. In F and J, circles represent the average terminal dendrite or spike length in a neuron. In D, F, I, and J, horizontal lines indicate the mean, and small number labels within the graph indicate the number of neurons analyzed. Scale bar in A and G = 100μm. *, p<0.05; **, p<0.01; ***, p<0.001; n.s., not significant using Student’s t-test with Welch correction (D-F) or Mann-Whitney test (I-J).

## Description

Neurons modify their structure and connectivity throughout their development in response to shifting internal and environmental cues. These changes require cytoskeletal rearrangements, and many previous studies have identified cytoskeleton-associated proteins as mediators of structural adaptation and refinement (Ouzounidis et al., 2023). *Drosophila melanogaster* larval dendritic arborization neurons (da neurons) are a valuable model for investigating the role of cytoskeletal regulators in neuron morphogenesis due to their complex branching patterns and their accessibility for imaging. In embryos and larvae, four classes of abdominal da neurons extend dendrites across cutaneous receptive fields to detect distinct environmental stimuli (reviewed in Singhania & Grueber, 2014). Class IV da neurons (C4da) and class III da neurons (C3da) have the most complex arbors but differ in branching structure. C4da neurons adopt an ordered branching structure, where primary dendrites bifurcate to form higher-order branches. In contrast, C3da arbors have long primary branches decorated with short, unbranched filopodial extensions (“spikes”) that emerge roughly perpendicular to the parent branch ( Fig. 1A and 1G; Grueber et al., 2002). In both neuron subtypes, terminal dendritic processes exhibit dynamic extension and retraction during larval stages (Grueber et al., 2003; Tsubouchi et al., 2012).

The microtubule plus end-associated polymerase Mini Spindles *,* a homolog of *Xenopus* XMAP25, was previously identified as a positive regulator of dendrite branch morphogenesis in C4da neurons (Das et al., 2017; Misra et al., 2016) and dendrite pruning during metamorphosis (Tang et al., 2020). We sought to further characterize the role of *msps* in da neurons during larval growth by examining the effects of reduced expression. &nbsp;To knock down *msps* , we used the C4da-specific driver pickpocket-Gal4 (ppk-Gal4) or the C2/C3da-specific driver 1003.3-Gal4 to drive expression of two *msps* -interfering shRNAs (“ *msps ^RNAi-1^* ” and “ *msps ^RNAi-2^* ”) via an upstream activating sequence.

In wandering third instar larvae, loss of *msps* activity caused a decrease in the number of terminal branches within the dendrite arbor of the ddaC neuron, a C4da neuron positioned within the dorsal da neuron cluster ( Fig. 1B- D). This result was consistent with previously reported findings (Das et al., 2017; Misra et al., 2016; Tang et al., 2020). The severity of the effects varied between shRNAs, with *msps ^RNAi-1^* eliciting a stronger effect (37% reduction) and *msps ^RNAi-2^* eliciting a mild but statistically significant effect (9% reduction). When we compared the number of dendritic termini at 48h and 72h after egg laying (AEL) to our results from wandering third instar (112-120h AEL), we found that the number of termini increased at each time point in control neurons but remained constant for *msps ^RNAi-1^* neurons ( Fig. 1E ). These findings support the conclusion that Msps is essential for the addition and/or maintenance of new branches during the period of “scaling growth”, 48h-120h AEL, when neuron arbors expand so their receptive fields can keep up with increasing larval surface area (Parrish et al., 2009). Previous studies have identified Msps as a regulator of acentrosomal microtubule nucleation (Deng et al., 2021); others have found that acentrosomal microtubule nucleation is essential for dendrite branch growth and stabilization (Ori-McKenney et al., 2012). Msps may be part of the molecular infrastructure that links these processes.

We also observed that *msps ^RNAi-1^* and *msps ^RNAi-2^* C4da neurons occasionally exhibited very long terminal branches. The average terminal dendrite length was significantly higher in *msps ^RNAi-2^* neurons than control (23% increase; Fig. 1F ). This was not true in *msps ^RNAi-1^* neurons; however, the effect may have been masked by severely depleted arbors overall. Together, our results suggest that Msps may function to promote the initiation of higher order dendrite branches while simultaneously protecting against excessive growth or erroneous maintenance of existing dendrites.

To determine if Msps plays a similar role in other neuronal subtypes, we examined the impact of knocking down *msps* expression in C3da neurons. As in C4da neurons, expression of the shRNA *msps ^RNAi-1^* caused a reduction in the total number of branches – in this case, spike-like filopodia – in the dorsal class III neuron ddaA. This resulted in a decrease in spike density (29% reduction; Fig. 1G- H, I). In addition, the average length of spikes increased in *msps ^RNAi-1^* ddaA neurons, echoing earlier observations in dorsal C4da neurons (33% increase; Fig. 1G- H, J). Further investigations will be required to confirm these results in *msps ^RNAi-2^* neurons. Of note, previous studies found that the distribution of cytoskeletal components varies in these two da neuron subtypes (Andersen et al., 2005; Tsubouchi et al., 2012). In C4da neurons, microtubules are present throughout the arbor; in C3da neurons, however, the filopodial spikes that protrude from primary dendritic branches are notably actin-rich and microtubule-poor. Our observations suggest that microtubule-associated proteins like Msps may significantly impact the growth and stability of these actin-based structures despite their relatively low microtubule content.

In conclusion, our findings suggest that the microtubule plus-end polymerase Mini Spindles promotes branch initiation and limits terminal dendrite growth in both C4da and C3da neurons during the period of scaling growth in *Drosophila* larvae. It is yet unclear whether Msps restricts terminal dendrite length by preventing excessive growth or by inhibiting maintenance. Tang and colleagues (2020) found that loss of *msps* function led to excessive maintenance of dendrites and a failure of pruning in C4da neurons during metamorphosis. They attributed this change in stability to the randomization of microtubule orientation in dendrites, a phenomenon that has also been linked to defects in branching (Sears & Broihier, 2016). Future studies may investigate whether Msps protects against excessive maintenance and stabilization of terminal dendrites to enable dendrite flexibility for growth, retraction, and branch initiation during the dynamic process of scaling neuron growth in *Drosophila* larvae.

&nbsp;

## Methods

***Drosophila* strains and genetics**

*D. melanogaster* stocks were reared on Nutri-Fly Instant or Nutri-Fly Bloomington Formulation media. Crosses were performed at 25°C for analysis of C4da neurons or at 29°C for analysis of C3da neurons. The following stocks were used: *ppk-GAL4* , *UAS-CD4-GFP* and *1003.3-GAL4; UAS-mCD8-GFP* (shared by Elizabeth Gavis, Princeton University); UAS-msps ^HMS01906^ (“UAS-msps ^RNAi-1^ ” in the text) and UAS-msps ^JF01613^ (“UAS-msps ^RNAi-2^ ” in the text) (Bloomington *Drosophila* Stock Center). In all experiments, *yw* flies served as the control.

**Microscopy**

Images were obtained using 20X/0.7 NA air and 40X/1.23 NA oil objectives on a Zeiss LSM710. For live imaging of GFP-expressing neurons, an imaging chamber was created by adhering two small (22x22mm) coverslips 5-7mm apart on a glass slide. Larvae were mounted between the two coverslips in a 1:2:2 mix of chloroform, halocarbon 95, and halocarbon 200. A long (24x50mm) coverslip was placed on top of the sample and held in place with laboratory tape. Dorsal C3da (ddaA and ddaF) or C4da (ddaC) neurons were imaged from abdominal segments 2 and 3. Neurons were imaged using tiled capture and automatic stitching. Images of C3da neurons comprised four tiles with overall dimensions of 850x850 μm. Images of dorsal C4da neurons include two tiles with overall dimensions of 425x850 μm. For C4da neurons, the imaging field typically included the dorsal, anterior, and posterior edges of the dendritic arbor but not branches extending ventrally from the cell body.

**Quantitative image analysis**

All quantitative image analysis was carried out using ImageJ (v2.1.0/1.53c). The Cell Counting plugin was used to count and mark all dendrite termini within the imaging field. For C3da neurons, the total number of termini was divided by the length of the primary dendrites to obtain a measure of spike density. Dendrite length was measured using the NeuronJ plugin (Meijering et al., 2004). For Class IV neurons, all terminal dendrites dorsal to the cell body within the image field were measured&nbsp;on neuron ddaC. For Class III, all spikes from neuron ddaA within the image field were measured. A range of 7-19 neurons from 3-6 larvae were analyzed for each experiment.

## Reagents

**Table:** 

**Reagent Type**	**Description**	**Source**	**Identifiers**	**Reference**
*D. melanogaster* stock	ppk-GAL4, UAS-CD4:tdGFP ^VK0003^	Gift from Elizabeth Gavis, Princeton University	BDSC_35836 BDSC_32079	(Bhogal et al., 2016)
*D. melanogaster* stock	1003.3-GAL4; UAS-mCD8-GFP	Gift from Elizabeth Gavis, Princeton University	FBti0128079	(Hughes & Thomas, 2007; Lee & Luo, 1999)
*D. melanogaster* stock	UAS-CD4:tdGFP ^VK0003^	Bloomington Stock Center	BDSC_35836	(Han et al., 2011)
*D. melanogaster* stock	UAS-mspsRNAi ^HMS01906^	Bloomington Drosophila Stock Center (Transgenic RNAi Project)	BDSC_38990	(Misra et al., 2016; Das et al., 2017)
*D. melanogaster* stock	UAS-mspsRNAi ^JF01613^	Bloomington Drosophila Stock Center (Transgenic RNAi Project)	BDSC_31138	(Gao et al., 2024)
*D. melanogaster* stock	yw	Laboratory stock		

## References

[R1] Andersen Ryan, Li Yimei, Resseguie Mary, Brenman Jay E. (2005). Calcium/Calmodulin-Dependent Protein Kinase II Alters Structural Plasticity and Cytoskeletal Dynamics in
*Drosophila*. The Journal of Neuroscience.

[R2] Bhogal Balpreet, Plaza-Jennings Amara, Gavis Elizabeth R. (2016). Nanos-mediated repression of
*hid*
protects larval sensory neurons after a switch in sensitivity to apoptotic signals. Development.

[R3] Das Ravi, Bhattacharjee Shatabdi, Patel Atit A, Harris Jenna M, Bhattacharya Surajit, Letcher Jamin M, Clark Sarah G, Nanda Sumit, Iyer Eswar Prasad R, Ascoli Giorgio A, Cox Daniel N (2017). Dendritic Cytoskeletal Architecture Is Modulated by Combinatorial Transcriptional Regulation in
*Drosophila melanogaster*. Genetics.

[R4] Deng Qiannan, Tan Ye Sing, Chew Liang Yuh, Wang Hongyan (2021). Msps governs acentrosomal microtubule assembly and reactivation of quiescent neural stem cells. The EMBO Journal.

[R5] Gao Z, Huang E, Wang W, Xu L, Xu W, Zheng T, Rui M (2024). Patronin regulates presynaptic microtubule organization and neuromuscular junction development in Drosophila.. iScience.

[R6] Grueber Wesley B., Jan Lily Y., Jan Yuh Nung (2002). Tiling of the
*Drosophila*
epidermis by multidendritic sensory neurons. Development.

[R7] Grueber Wesley B., Ye Bing, Moore Adrian W., Jan Lily Y., Jan Yuh Nung (2003). Dendrites of Distinct Classes of Drosophila Sensory Neurons Show Different Capacities for Homotypic Repulsion. Current Biology.

[R8] Han Chun, Jan Lily Yeh, Jan Yuh-Nung (2011). Enhancer-driven membrane markers for analysis of nonautonomous mechanisms reveal neuron–glia interactions in
*Drosophila*. Proceedings of the National Academy of Sciences.

[R9] Hattori Yukako, Sugimura Kaoru, Uemura Tadashi (2007). Selective expression of Knot/Collier, a transcriptional regulator of the EBF/Olf‐1 family, endows the
*Drosophila*
sensory system with neuronal class‐specific elaborated dendritic patterns. Genes to Cells.

[R10] Hughes Cynthia L., Thomas John B. (2007). A sensory feedback circuit coordinates muscle activity in Drosophila. Molecular and Cellular Neuroscience.

[R11] Jinushi-Nakao Shiho, Arvind Ramanathan, Amikura Reiko, Kinameri Emi, Liu Andrew Winston, Moore Adrian Walton (2007). Knot/Collier and Cut Control Different Aspects of Dendrite Cytoskeleton and Synergize to Define Final Arbor Shape. Neuron.

[R12] Lee Tzumin, Luo Liqun (1999). Mosaic Analysis with a Repressible Cell Marker for Studies of Gene Function in Neuronal Morphogenesis. Neuron.

[R13] Meijering E., Jacob M., Sarria J.‐C.F., Steiner P., Hirling H., Unser M. (2004). Design and validation of a tool for neurite tracing and analysis in fluorescence microscopy images. Cytometry Part A.

[R14] Misra Mala, Edmund Hendia, Ennis Darragh, Schlueter Marissa A, Marot Jessica E, Tambasco Janet, Barlow Ida, Sigurbjornsdottir Sara, Mathew Renjith, Vallés Ana Maria, Wojciech Waldemar, Roth Siegfried, Davis Ilan, Leptin Maria, Gavis Elizabeth R (2016). A Genome-Wide Screen for Dendritically Localized RNAs Identifies Genes Required for Dendrite Morphogenesis. G3 Genes|Genomes|Genetics.

[R15] Ori-McKenney Kassandra M., Jan Lily Yeh, Jan Yuh-Nung (2012). Golgi Outposts Shape Dendrite Morphology by Functioning as Sites of Acentrosomal Microtubule Nucleation in Neurons. Neuron.

[R16] Ouzounidis Vasileios R., Prevo Bram, Cheerambathur Dhanya K. (2023). Sculpting the dendritic landscape: Actin, microtubules, and the art of arborization. Current Opinion in Cell Biology.

[R17] Parrish Jay Z., Xu Peizhang, Kim Charles C., Jan Lily Yeh, Jan Yuh Nung (2009). The microRNA bantam Functions in Epithelial Cells to Regulate Scaling Growth of Dendrite Arbors in Drosophila Sensory Neurons. Neuron.

[R18] Sears James C., Broihier Heather T. (2016). FoxO regulates microtubule dynamics and polarity to promote dendrite branching in Drosophila sensory neurons. Developmental Biology.

[R19] Singhania Aditi, Grueber Wesley B. (2014). Development of the embryonic and larval peripheral nervous system of
*Drosophila*. WIREs Developmental Biology.

[R20] Tang Quan, Rui Menglong, Bu Shufeng, Wang Yan, Chew Liang Yuh, Yu Fengwei (2020). A microtubule polymerase is required for microtubule orientation and dendrite pruning in
*Drosophila*. The EMBO Journal.

[R21] Tsubouchi Asako, Caldwell Jason C., Tracey W. Daniel (2012). Dendritic Filopodia, Ripped Pocket, NOMPC, and NMDARs Contribute to the Sense of Touch in Drosophila Larvae. Current Biology.

